# A pilot study on the efficacy of topical lotion containing anti-acne postbiotic in subjects with mild -to -moderate acne

**DOI:** 10.3389/fmed.2022.1064460

**Published:** 2022-12-09

**Authors:** Hongchang Cui, Chaoqun Guo, Qian Wang, Congrui Feng, Zhi Duan

**Affiliations:** Qingdao Vland Biotech Group Co., Ltd., Qingdao, China

**Keywords:** acne, postbiotic, probiotic, *Lactiplantibacillus plantarum*, topical

## Abstract

**Introduction:**

Acne can compromise facial esthetics and become a mental burden, especially when it occurs in puberty. Skincare cosmetics with anti-acne efficiency is more convenient than other treatment modalities, such as dietary supplements, in certain circumstances. The purpose of this study was to investigate the efficacy of an anti-acne lotion in alleviating acne.

**Methods:**

In our study, an anti-acne lotion containing ferment lysate produced by *Lactiplantibacillus plantarum* VHProbi^®^ E15 were applied to subjects with mild -to -moderate acne over 4 weeks. The efficacy was evaluated based on instrumental measurements using Visia^®^-CR and CK-MPA^®^ system.

**Results and discussion:**

The anti-acne lotion exhibited favorable safety, meeting the stringent criteria for the detection of microbes, heavy metals, toxicity, and irritation. After 2 weeks of treatment, a statistically significant improvement in acne lesions was observed compared to baseline (*P* < 0.01), and this continued to the end of the study. After 4 weeks of treatment, the transepidermal water loss (*P* < 0.05) and sebum production (*P* < 0.05) were significantly decreased in subjects compared to baseline. In addition, the pore/area of interest (AOI) and stratum corneum hydration displayed slightly positive changes throughout treatment. Thus, we conclude that applying topical anti-acne lotion may be safe and confer effective benefits in people with mild -to -moderate acne and represents a promising therapeutic option for acne.

## Introduction

Acne is a chronic inflammatory disorder involving pilosebaceous units and is the eighth most prevalent cutaneous disease, typically ranging from mild lesions such as comedones to more severe conditions including papules, pustules, and nodules (1, 2). Additionally, symptoms such as hyperpigmentation, erythema, and scars are usually associated with the presence of acne (3). The appearance of acne is highly associated with a higher density of sebaceous glands in the face, shoulders, neck, and back (4). In the past few decades, numerous studies have shown that there are 4 pivotal processes involved the formation of acne lesions: the release of inflammatory cytokines into the skin, an altered keratinization process leading to comedones, increased and altered sebum secretion under androgen control, and follicular colonization by *Cutibacterium acnes* (5). Based on the Global Burden Disease study in 204 countries, the incidence of acne vulgaris is fairly high, affecting more than 231 million people across genders and causing the loss of 4.96 million (95% UI 2.98–7.85) disability adjusted life years in 2019 (6).

Acne usually begins at puberty as the sexual organs start to physically mature and become aggravated in adolescence, a crucial time for building confidence (7). Furthermore, a severe acne can have a negative effect on an individual’s social interactions and self-esteem, with accompanying psychological issues such as shame, embarrassment, anxiety, depression, and social inhibition (4). Anger negatively correlates with quality of life in acne and satisfaction with acne treatments (8). Different treatment guidelines involve approximately similar approaches, aiming to help individuals to improve their better appearance and prevent mental sequelae (9, 10).

In general, topical agents such as benzoyl peroxide, retinoids, antibiotics, and others are used alone for mild -to -moderate acne or in combination therapy for severe acne (11–13). Oral treatments such as antibiotics, contraceptives, and isotretinoin are usually reserved for severe acne or acne that is unresponsive to topical therapies (4, 14, 15). The use of complementary and alternative medical treatments for acne, including the Kampo, tea tree oil products, pyridoxine, and fruit-derived acid is quite popular in some cultures (16, 17). However, their application in the treatment of acne has been limited by their costs, individual preference and their side effects. For instance, the overuse of oral and topical antibiotics results in deteriorated bacterial resistance, especially when they are used for a long time at lower doses (18, 19). Concerns regarding the disadvantages of existing therapies have increased the urgency of developing novel therapies.

A joint report by the Food and Agriculture of Organization of the United Nations and the World Health Organization (WHO) on the health and nutritional properties of probiotics in food defined probiotics as “*live microorganisms which when administrated in adequate amounts can confer a health benefit on the host*” (20). Numerous clinical studies have illustrated the positive effects of probiotics on the gut, nerves, metabolism, immunity, etc., such as in inflammatory bowel disease, depression, hypercholesterolemia, and respiratory disease (21–24). Emerging evidence from interdisciplinary research has elucidated the existence of communication between the gut and the skin, usually called the gut - skin axis (25, 26). There is rapidly growing interest in the administration of probiotics for anti-ageing treatment and the treatment of atopic eczema and acne (9, 27, 28). The beneficial effects of probiotic strains are very specific, which means that not all strains of the same species can deliver health benefits against the particular diseases (29). Meanwhile, the direct use of live microorganisms as functional ingredients in cosmetic products is prohibited in China, which means that alternative methods are needed to meet the legal requirements.

Recently, a variety of preclinical and clinical studies have demonstrated that postbiotics, which are preparations of inanimate microorganisms and/or their components, elicit health-promoting effects (30). These findings highlight a new generation of dietary supplements, or nutraceuticals, which could be safer alternatives for clinical applications, especially in cutaneous disorders such as acne, where the use of probiotics may not be legal. In our previous study, a strain of *Lactiplantibacillus plantarum* VHProbi^®^ E15 was isolated from Kimchi soup, which showed antagonism to the *Cutibacterium acnes in vitro*. Here, we investigated whether postbiotic preparations produced by *L. plantarum* VHProbi^®^ E15 could ameliorate the severity of subjects with mild -to -moderate acne.

## Materials and methods

### *In vitro* studies

#### Bacterial strains

Samples of kimchi soup were used to inoculated de Man-Rogosa-Sharpe (MRS) agar plate medium (cat. No.: HB0384, Hope Bio-Technology Co., Ltd) and incubated under aerobic conditions at 37°C for 72 h (31). A lactic acid bacterium was isolated and designated as *L. plantarum* VHProbi^®^ E15 based on polyphasic analysis (32). *C*. *acnes* ATCC 11827 and ATCC 6919 were purchased from American Type Culture Collection, (Manassas, VA, USA). *C. acnes* ATCC 11827 and ATCC 6919 were maintained on a Reinforced Clostridial Medium (RCM, cat. no.: HB0316, Hope Bio-Technology Co., Ltd) and incubated in an anaerobic jar (Sigma-Aldrich^®^) with an oxygen absorber CO_2_ generator (AnaeroPack C-1, MGC^®^, Japan) at 37°C for 48 h.

#### Antimicrobial activity of *Lactiplantibacillus plantarum* VHProbi^®^ E15

The inhibitory activity of *L. plantarum* VHProbi^®^ E15 against *C. acnes* ATCC 11827 and ATCC 6919 was evaluated using a double-layer agar plate assay and agglutination test, as described previously with some modifications (33, 34). Briefly, bacterial spots were inoculated with 100 μL of test bacterium culture (10^9^ CFU/mL) in triplicate plus a blank on the RCM agar surface in a Petri dish and incubated at 37°C for 48 h. Then, the plate was overlaid with 8 mL of RCM soft agar (0.75%, 1% Tween 80) which had been inoculated with 10^6^ CFU/mL of mixed cultures of *C. acnes* ATCC 11827 and ATCC 6919 and incubated at 37°C for 72 h. The plate was examined for the presence or absence of an inhibitory halo around the spots. In the agglutination test, 300 μL of the test bacterium culture (10^9^CFU/mL) was co-incubated with 300 μL of mixed cultures of *C. acnes* ATCC 11827 and ATCC 6919 (10^6^ CFU/mL) in 24 -well plates at 37°C in a thermostatic oscillation incubator (HUXI^®^, HW-400TG). At the same time, the test bacterium culture was co-incubated with Tris -HCI buffer (Sigma), used as negative control simultaneously. After 24 h, the plates were then checked for the presence or absence of tiny white floccules.

### Clinical studies

#### Bacterial lysate

The fermentation medium contained 3% brown sugar, 3% collagen, 0.3% yeast extract, 0.25 % (NH_4_)_2_HPO_4_, and 1000 mL distilled water. Strains of *L. plantarum* VHProbi^®^ E15 were prepared for use by subculturing in MRS broth at 37°C for 24–48 h no fewer than 2 but no more than 7 times. Afterward, the seed cultures were used to inoculated a fermentation medium with 1% (w/v) and incubated under aerobic conditions at 37°C for 48 h. The fermentation cultures were homogenized under 1000 bar using a homogenizer device (SPXFLOW, APV-1000); then, the lysate was harvested as the functional ingredient after being heated at 70°C for 15 min.

#### Anti-acne lotion

The content of the anti-acne lotion was as follows: 8% ferment lysate, 0.4% cetearyl alcohol, 0.05% EDTA-2Na, 0.35% polyacrylate crosspolymer –6, 3% caprylic, 4% glycerin, 0.8% butyrospermum parkii, 0.4% 1,2 -hexanediol, 1.5% cetearyl olivate, 0.6% sorbitan olivate, 0.3% hyroxyacetophenone, 2.5% isohexadecane, 1% PDMS, 0.2% phenoxyethanol. These ingredients, except for ferment lysate, function as chelating agents, thickeners, and preservatives in the lotion. This formulation was planned and revised based on trial products provided by YISU Biotech Ltd.

#### Safety assay

Safety assessments were prepared based on the Safety and Technical Standards of Cosmetics (2015 version) issued by the National Medicine Product Administration in China (35). Toxicological tests, microbial tests, and physical and chemical inspections were commissioned to the Health Analysis and Test Center of Nanjing Medical University. Specifically, the detection of heavy metals, including Cr, Hg, As, and Pb were performed according to the method named atomic absorption spectrophotometer (36). An acute transepidermal toxicity tests and skin irritation patch tests were commissioned to Pony Test International Group.

#### Subjects

In total, 22 male and female adult subjects were recruited after verification of the study’s inclusion and exclusion criteria (Table 1) at the Cosmetic Innovation Center of Jiangnan University in Jiangsu province, China. All the subjects were informed the purpose of this study and provided informed written consent. Ethic approval was not required in accordance with the Safety and Technology of Standards for Cosmetics (37). The research has complied with all the relevant federal guidelines and institutional policies. Only subjects with mild -to -moderated acne, with scores of 2 or 3 on the Global Acne Assessment Scale (Table 2), were included (38). The demographic information of all subjects was presented in the Supplementary Table 1.

**TABLE 1:** Inclusion/exclusion criteria.

Inclusion criteria	Exclusion criteria
Male or female > 16 years old	Known sensitivity to any compound of the investigated products
Signed informed consent form	Pregnant or breastfeeding
No participation in similar study currently or during previous 6 months	Facial disease or having any facial cosmetic procedures within 3 months
Ability to follow the instructions from investigators and return to study center at established time	History of cosmetic product use within a fortnight that, in investigator’s opinion, could interfere with assessment
Last treatment was at least 2 months ago	Significant psychological and psychiatric disorders that could impair subjects’ ability to meet study requirements
Non responsive to previous treatments, either systemic, topical, or phototherapy	History of concurrent malignancy
Not receiving any drugs/cosmetic treatments that might interfere with current study	Any medical condition that, in investigator’s opinions, could prevent the subjects from participating in study

**TABLE 2:** Global acne assessment scale.

Grade	Description
0	Normal, clear skin with no evidence of acne
1	Skin is almost clear, rare inflammatory lesions present, with rare non-inflamed papules (papules must be resolving and maybe hyperpigmented, although not pink-red), requiring no further treatment in investigator’s opinion
2	Some noninflammatory lesions present, with few inflammatory lesions (papules/pustules only; no nodulocystic lesions)
3	Noninflammatory lesions predominate, with multiple inflammatory lesions evident; several to many comedones and papules/pustules, and there may or may not be one small nodulocystic lesion
4	Inflammatory lesions are apparent; many comedones and papules/pustules; there may or may not be a few nodulocystic lesions
5	Highly inflammatory lesions predominate; variable number of comedones; many papules/pustules and nodulocystic lesions

#### Study design and outcome measurements

The study was conducted for 4 weeks. The subjects applied an appropriate amount of the anti-acne lotion twice daily to their foreheads, jaws, noses, and cheeks after cleansing their faces. Before being evaluated, the subjects were asked to rest for 20 min in an assessment room at 21 ± 1°C, with a relative humidity of 50 ± 10%. The dermatology assessments, including those of acne lesion proportion and pore/area of interest (AOI) (mm^2^), were performed using a Visia^®^-CR system (Canfield Scientific Inc.) at baseline and during follow-up visits at Weeks 1, 2, 3, and 4 after treatment initiation. Other clinical evaluations, including for the stratum corneum hydration (SCH), transepidermal water loss (TEWL), sebum contents (SC) and skin superficial pH, were performed using a CK-MPA^®^ system (CK-MPA10, Courage + Khazaka electronic GmbH) at baseline and during follow-up visits at Weeks 2 and 4 after treatment initiation.

#### Statistical analysis

The Shapiro-Wilk test was used to determine the normality of the data distribution. Parametric values are expressed as means ± standard deviations/standard errors, nonparametric values are expressed as percentages. Single-factor repeated measures ANOVA was used for efficacy assessment between the visit times, and all the statistical analyses were performed using SPSS ver. 20.0 (SPSS Inc., Chicago, IL, USA). The statistical tests used a significance level of α ≤ 0.05. The improvement rates (IRs) for different indicators between baseline and each follow-up visit were calculated by using the following formula:


$$\%\mathrm{IR}\text{s}=\frac{{\text{W}}_{\text{i}}-{\text{W}}_{0}}{{\text{W}}_{0}}\times 100\%$$

where W_*i*_ is the assessment value at Week i (i = 1, 2, 3, or 4), and W_0_ is the assessment value at baseline. The boxplots were created by imageGP service (39).

## Results

### *In vitro* microbial study

**FIGURE 1:**
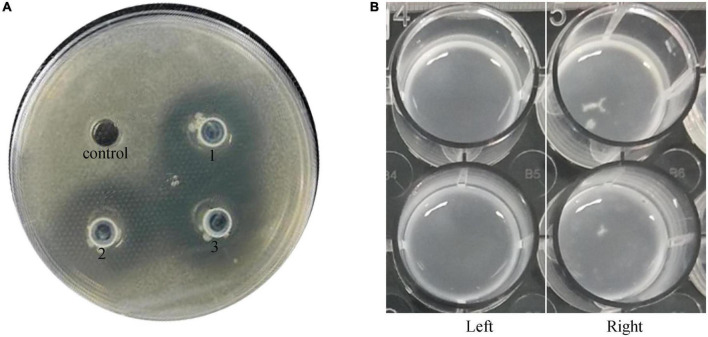
Microbial activity of investigated strain. **(A)** Double-layer plate assay: control: blank; experimental group: 1, 2, and 3. **(B)** Agglutination test: left, blank; right, experimental group.

The double-layer plate assay showed an unambiguous halo around the spots in the plate with a diameter of 29.00 ± 1.00 mm (Figure 1A). This indicated that the bacterial strain *L. plantarum* VHProbi^®^ E15 can inhibit the growth of *C. acnes*. In the agglutination assay, the *L. plantarum* VHProbi^®^ E15 did not show the self-agglutination after 72 h of incubation (Figure 1B, left). The first time of observation of tiny white floccule was 30 h 10 min and the floccules began to aggregate in the co-incubation wells after 48 h (Figure 1B, right).

### Clinical studies

#### Safety assessment

A cutaneous irritation test was conducted using 4 New Zealand white rabbits; the anti-acne lotion was applied to their back skin once daily for 14 days. None of the rabbits exhibited erythema or edema during the treatment. The enumeration of the total microbial colonies, mycete and yeast colonies, thermotolerant coliform, *Staphylococcus aureus*, and *Pseudomonas aeruginosa* was performed using the agar plate method (40). All the microbial counts were lower than the detection limit. Likewise, physical and chemical inspections showed that the contents of Cr, Hg, As, and Pb were lower than the detection limit. Acute transepidermal toxicity tests were performed using specific pathogen free rats. The results show that the median lethal dose (LD_50_) of the anti-acne lotion in rats exceeded 2180 mg/kg body weight (BW), which means the lotion can be graded as low-poison preparation. The result from the skin irritation tests showed that none of the 30 subjects, who participated in the 24-h patch test, had undesirable skin irritation reactions to the anti-acne lotion.

#### Acne lesion proportion

**FIGURE 2:**
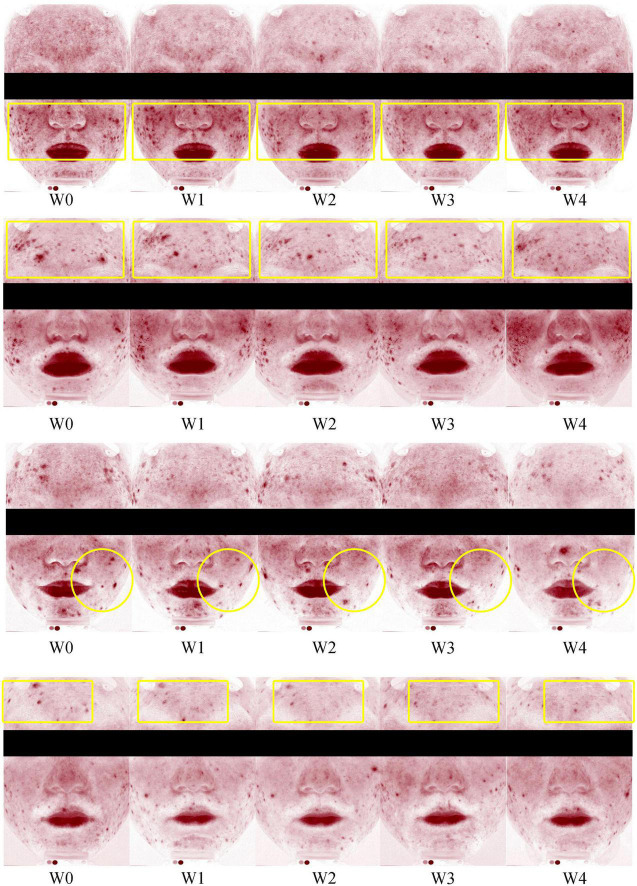
Representative photographic evaluation of improvements in acne lesions at Weeks 0 (W0), 1 (W1), 2 (W2), 3 (W4), and 4 (W4).

**FIGURE 3:**
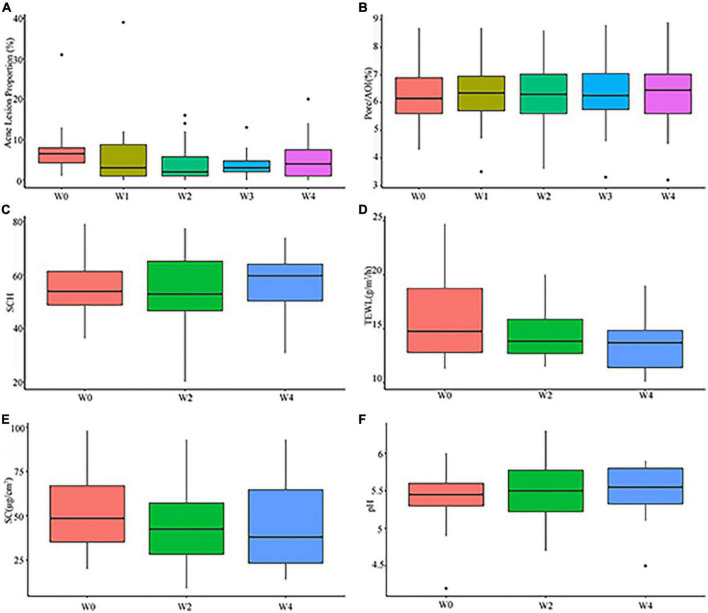
Clinical assessment of subjects at Weeks 0 (W0),1 (W1), 2 (W2), 3 (W3), and 4 (W4): **(A)** proportions of acne lesion; **(B)** pore/AOI; **(C)** stratum corneum hydration (SCH); **(D)** transepidermal water loss (TEWL); **(E)** sebum contents (SC); **(F)** pH.

The percentage decrease in acne lesions from baseline to each follow-up visit is presented in Supplementary Table 2. A representative photographic assessment of subjects with acne lesions is shown in Figure 2. At Week 2, there was a statistically significant decrease in the proportion of acne lesion compared to the baseline (*P* < 0.01) (see Figure 3A). At Weeks 3 and 4, there was a consistent and high level of significant improvement in the proportions of acne lesion (*P* < 0.01, *P* < 0.01, respectively). Among the subjects, 59.1% of subjects had a decreased proportion of acne lesion and 61.5 % of them had a decrease of over 50 % at Week 4.

#### Pore/Area of interest (mm^2^)

The results of evaluating the pore/AOI (mm^2^) from baseline to each follow-up visit are presented in Supplementary Table 3. In the pore/AOI assessment, lower scores indicate few pores. There was no significant difference from baseline to each follow-up visit (*P* > 0.05) (see Figure 3B), although 31.8% of the subjects showed a slight decrease in pore/AOI (mm^2^) at Week 4.

#### Stratum corneum hydration

The results of evaluating the SCH from baseline to each follow-up visit are presented in Supplementary Table 4. Higher scores mean high skin moisture contents. Among subjects, 36.4 and 59.1 % of subjects had improved in SCH at Weeks 2 and 4, respectively. However, the increases were not statistically significant compared to baseline (*P* > 0.05) (see Figure 3C).

#### Transepidermal water loss

The results of accessing the TEWL from baseline to each follow-up visit are presented in Supplementary Table 5. Lower scores indicate better barrier function in the skin. At Week 2, 63.6 % of the subjects had significantly improved TEWL, but there was no statistical significance compared to baseline (*P* > 0.05). At Week 4, 68.2 % of the subjects had significantly improved TEWL compared to baseline (*P* < 0.05), and 66.7 % of these subjects had reductions greater than 20 % (see Figure 3D).

#### Sebum contents

The results of accessing the sebum content from baseline to each follow-up visit are presented in Supplementary Table 6. Lower scores indicate lower sebum secretion in the skin. The statistical results showed a significant degree of improvement at each follow-up visit compared to baseline (P < 0.05, P < 0.05, respectively) (see Figure 3E). At Week 4, 72.7 % of the subjects had a lower sebum production compared to baseline.

#### Facial skin pH

The results of accessing the skin pH from baseline to each follow-up visit are presented in Supplementary Table 7. Normal skin pH is close to weakly acidic. The subjects showed relatively steady facial pH at each follow-up visit, and no significant difference was observed compared to baseline (*P* > 0.05 and, *P* > 0.05, respectively) (see Figure 3F).

## Discussion

Acne vulgaris is one of the most common forms of dermatosis related to sebum glands and hair follicles and occurred worldwide, causing severe disfiguring scars and even psychological problems (9). Patients usually fail to adhere to the treatment due to the need for long-term use, side effects, and expenses (41–43). The field of acne treatments hesitated to move forward for many years, and there is clearly a compelling need for safer and more effective therapy with fewer complications (44).

An expanding body of research has highlighted that the interaction between the skin microbiota and host immunity plays an important role in many cutaneous diseases (45). Dysbiosis in the skin microbiome has commonly been found in acne patients, with increased richness of *C. acnes* in lipid-rich environments (46). It is increasingly believed that the over-colonization of certain ribotypes of *C. acnes* contributes to the pathophysiology of acne (47). Some acne-related *C. acnes* strains can lead to inflammatory responses in keratinocytes, sebocytes, and peripheral blood mononuclear cells (48–51). Lebeer et al. substantiated that selected and formulated lactobacilli can reduce inflammatory lesions in patients with mild -to -moderate acne, resulting a reduction in the relative abundance of *Staphylococci spp.* and *C. acnes* (52). Rinaldi et al. tested the efficacy of a dietary supplement containing probiotics in subjects with mild -to -moderate acne and found that the relative abundance of *C. acnes* and *Staphylococcus aureus* was significantly decreased after 4 and 8 weeks of treatment compared to baseline (53).

Postbiotics are bioactive substances that are secreted in a medium or can be harvested through cell rupture, including soluble factors and metabolic by-products. Lew et al. confirmed that bacteriocins, one of the most common microbial agents, can inhibit the growth of *C. acnes* and regulate the inflammatory response in the epidermis and dermis (54, 55). Kang et al. used enterocins from *Enterococcus faecalis* SL-5 as a postbiotic to treat patients with mild -to -moderated acne lesions, and found that inflammatory lesions and pustules were significantly decreased compared to placebo (56). Other examples of postbiotics include organic acids and fatty acids. Both of these extracellular metabolites have been shown to exhibit microbial activity in skin tissue. Fatty acids can also prevent TEWL in the stratum corneum (57, 58).

In the present study, ferment lysate produced by *L. plantarum* VHProbi^®^ E15 was used as a postbiotic to treat patients with mild -to -moderate acne. First, *L. plantarum* VHProbi^®^ E15 showed apparent antagonism against *C. acnes in vitro*, confirming a direct competitive exclusion effect among probiotic and acne-associated bacteria. A formulated lotion containing ferment lysate was shown to have a good safety profile in several assessments, and no side effects related to the lotion occurred during the research. In the pilot study, significant improvements in acne lesions, TEWL, and SC were observed after 4 weeks of treatment. The numbers of acne lesion may not indicate clearly diminished trend because of the emergence of some tiny and indistinct lesions. However, the proportions of acne lesion in the entire face declined in comparison with baseline. Although the indicators of pore/AOI and SCH did not show significantly meaningful improvement at Week 4 compared to baseline, a positive trend after treatment could be observed. We speculate that the ferment lysate of *L. plantarum* VHProbi^®^ E15 contains microbial substances that contributes to its favorable effect on ameliorating inflammatory acne lesions and protecting skin barrier integrity.

Using probiotics as an ingredient in topical formulations poses a variety of challenges, such as maintaining the viability of bacteria and higher complexity in manufacturing, not to mention the legal issues in China. Conversely, postbiotics can be used as a preferred ingredient in cosmetics because they involve less complicated storage conditions and easier selection of preservative ingredients. Given these aspects, this bioactive ingredient may have the potential to be a novel safe and effective therapy for mild -to -moderate acne. However, there were some limitations in this study: no elucidation of the exact functional components in ferment lysate, the relatively small population, and the short-term nature of the intervention.

## Conclusion

The results from this study suggested a topical anti-acne skincare lotion containing ferment lysate from *L. plantarum* VHProbi^®^ E15 in people with mild -to -moderate acne is safe and well tolerated and may be able to ameliorate acne lesions after 4 weeks of treatment. Furthermore, the topical postbiotic lotion was shown to improve moisturization and control skin pH, which are positive outcomes in acne treatment. Detailed molecular studies are needed to determine the underlying immunomodulatory mechanism related to postbiotic components, that contribute to this novel skincare method based on microbiome modulation.

## Data availability statement

The original contributions presented in this study are included in the article/Supplementary material, further inquiries can be directed to the corresponding author.

## Ethics statement

Ethical review and approval was not required for the study on human participants in accordance with the local legislation and institutional requirements. The patients/participants provided their written informed consent to participate in this study. The animal study was reviewed and approved by Jiangnan University. Written informed consent was obtained from the individual(s) for the publication of any potentially identifiable images or data included in this article.

## Author contributions

All authors listed have made a substantial, direct, and intellectual contribution to the work, and approved it for publication.

## References

[1] Hay R Johns N Williams H Bolliger I Dellavalle R Margolis D The global burden of skin disease in 2010: an analysis of the prevalence and impact of skin conditions. *J Invest Dermatol.* (2014) 134:1527–34. 10.1038/jid.2013.446 24166134

[2] Degitz K Placzek M Borelli C Plewig G. Pathophysiology of acne. *J Dtsch Dermatol Ges.* (2007) 5:316–23. 10.1111/j.1610-0387.2007.06274.x 17376098

[3] Mahto A. Acne vulgaris. *Medicine (Baltimore).* (2017) 45:386–9. 10.1016/j.mpmed.2017.03.003

[4] Williams H Dellavalle R Garner S. Acne vulgaris. *Lancet.* (2012) 379:361–72. 10.1016/S0140-6736(11)60321-8 21880356

[5] Thiboutot D Gollnick H Bettoli V Dréno B Kang S Leyden J New insights into the management of acne: an update from the global alliance to improve outcomes in acne group. *J Am Acad Dermatol.* (2009) 60:S1–50. 10.1016/j.jaad.2009.01.019 19376456

[6] Global Health Metrics. *Acne Vulgaris– Level 3.* Cleveland, OH: Global Health Metrics (2019).

[7] Tan J Bhate K. A global perspective on the epidemiology of acne. *Br J Dermatol.* (2015) 172:3–12. 10.1111/bjd.13462 25597339

[8] Rapp D Brenes G Feldman S Fleischer A Jr Graham G Dailey M Anger and acne: implications for quality of life, patient satisfaction and clinical care. *Br J Dermatol.* (2004) 151:183–9. 10.1111/j.1365-2133.2004.06078.x 15270889

[9] Leung A Barankin B Lam J Leong K Hon K. Dermatology: how to manage acne vulgaris. *Drugs Context.* (2020) 10:1–18. 10.7573/dic.2021-8-6 34691199 PMC8510514

[10] Zaenglein A Pathy A Schlosser B Alikhan A Baldwin H Berson D Guidelines of care for the management of acne vulgaris. *J Am Acad Dermatol.* (2016) 74:945–73.e33. 10.1016/j.jaad.2015.12.037 26897386

[11] Asai Y Baibergenova A Dutil M Humphrey S Hull P Lynde C Management of acne: Canadian clinical practice guideline. *CMAJ.* (2016) 188:118–26. 10.1503/cmaj.140665 26573753 PMC4732962

[12] Otlewska A Baran W Batycka-Baran A. Adverse events related to topical drug treatments for acne vulgaris. *Expert Opin Drug Saf.* (2020) 19:513–21. 32347138 10.1080/14740338.2020.1757646

[13] Basak S Zaenglein A. Acne and its management. *Pediatr Rev.* (2013) 34:479–97. 10.1542/pir.34.11.479 24187141

[14] Leung A Barankin B Hon K. Adolescent acne vulgaris: an overview of therapeutic options. *Consultant Pediatr.* (2015) 14:63–5.

[15] Roman C Cifu A Stein S. Management of acne vulgaris. *JAMA.* (2016) 316:1402–3. 10.1001/jama.2016.11842 27701644

[16] Magin P Adams J Pond C Smith W. Topical and oral CAM in acne: a review of the empirical evidence and a consideration of its context. *Complement Ther Med.* (2006) 14:62–76. 10.1016/j.ctim.2005.10.007 16473756

[17] Hammer K. Treatment of acne with tea tree oil (Melaleuca) products: a review of efficacy, tolerability and potential modes of action. *Int J Antimicrob Agents.* (2015) 45:106–10. 10.1016/j.ijantimicag.2014.10.011 25465857

[18] Eady A Cove J Layton A. Is antibiotic resistance in cutaneous propionibacteria clinically relevant? *Am J Clin Dermatol.* (2003) 4:813–31. 10.2165/00128071-200304120-00002 14640775

[19] Høiby N Jarløv J Kemp M Tvede M Bangsborg J Kjerulf A Excretion of ciprofloxacin in sweat and multiresistant *Staphylococcus epidermidis*. *Lancet.* (1997) 349:167–9. 10.1016/S0140-6736(96)09229-X 9111541

[20] Food and Health Agricultural Organisation of the United Nations, World Health Organisation. *Guidelines for the Evaluation of Probiotics in Food. Working Group Report.* Rome: Food and Health Agricultural Organisation of the United Nations, World Health Organisation (2002). p. 1–11.

[21] Jakubczyk D Leszczyńska K Górska S. The effectiveness of probiotics in the treatment of inflammatory bowel disease (IBD)—a critical review. *Nutrients.* (2020) 12:1973. 10.3390/nu12071973 32630805 PMC7400428

[22] Sivamaruthi B Kesika P Chaiyasut C. A mini-review of human studies on cholesterol-lowering properties of probiotics. *Sci Pharm.* (2019) 87:26. 10.3390/scipharm87040026

[23] Nikolova V Zaidi S Young A Cleare A Stone J. Gut feeling: randomized controlled trials of probiotics for the treatment of clinical depression: systematic review and meta-analysis. *Ther Adv Psychopharmacol.* (2019) 9:2045125319859963. 10.1177/2045125319859963 31263542 PMC6595633

[24] Shahbazi R Yasavoli-Sharahi H Alsadi N Ismail N Matar C. Probiotics in treatment of viral respiratory infections and neuroinflammatory disorders. *Molecules.* (2020) 25:4891. 10.3390/molecules25214891 33105830 PMC7660077

[25] Lee Y Byun E Kim H. Potential role of the microbiome in acne: a comprehensive review. *J Clin Med.* (2019) 8:1–25. 10.3390/jcm8070987 31284694 PMC6678709

[26] Szyguła R Asanova B Chilicka K Dzie I. Microbiome and probiotics in acne vulgaris— A narrative review. *Life (Basel).* (2022) 12:422. 10.3390/life12030422 35330173 PMC8953587

[27] Levkovich T Poutahidis T Smillie C Varian B Ibrahim Y Lakritz J Probiotic bacteria induce a “glow of health.”. *PLoS One.* (2013) 8:e53867. 10.1371/journal.pone.0053867 23342023 PMC3547054

[28] Mottin V Suyenaga E. An approach on the potential use of probiotics in the treatment of skin conditions: acne and atopic dermatitis. *Int J Dermatol.* (2018) 57:1425–32. 10.1111/ijd.13972 29676446

[29] Roudsari MR Karimi R Sohrabvandi S Mortazavian AM. Health effects of probiotics on the skin. *Crit Rev Food Sci Nutr.* (2015) 55:1219–40. 10.1080/10408398.2012.680078 24364369

[30] Salminen S Collado M Endo A Hill C Lebeer S Quigley E The international scientific association of probiotics and prebiotics (ISAPP) consensus statement on the definition and scope of postbiotics. *Nat Rev Gastroenterol Hepatol.* (2021) 18:649–67. 10.1038/s41575-021-00440-6 33948025 PMC8387231

[31] Lee K Shim J Park S Heo H Kim H Ham K Isolation of lactic acid bacteria with probiotic potentials from kimchi, traditional Korean fermented vegetable. *LWT Food Sci Technol.* (2016) 71:130–7. 10.1016/j.lwt.2016.03.029

[32] Jia C Cui H Han Y Fu T Du R Wang X *Ancylomarina psychrotolerans* sp. nov., isolated from sediments of fildes Peninsula and emended the description of genus *Ancylomarina*. *Antonie Van Leeuwenhoek.* (2018) 111:1183–9. 10.1007/s10482-018-1025-9 29453612

[33] Damaceno Q Souza J Nicoli J Paula R Assis G Figueiredo H Evaluation of potential probiotics isolated from human milk and colostrum. *Probiotics Antimicrob Proteins.* (2017) 9:371–9. 10.1007/s12602-017-9270-1 28374172

[34] Ahn C Stiles M. Antibacterial activity of lactic acid bacteria isolated from vacuum-packaged meats. *J Appl Bacteriol.* (1990) 69:302–10. 10.1111/j.1365-2672.1990.tb01520.x 2123171

[35] National Medicine Products Administration. *Safety and Technical Standards for Cosmetics.* (2015). Available online at: https://www.nmpa.gov.cn/directory/web/nmpa/images/MjAxNcTqtdoyNji6xbmruOa4vbz+LnBkZg==.pdf (accessed February 18, 2022).

[36] Tüzen M. Determination of heavy metals in soil, mushroom and plant samples by atomic absorption spectrometry. *Microchem J.* (2003) 74:289–97. 10.1016/S0026-265X(03)00035-3

[37] National Medicine Products Administration. *Safety and Technology of Standards for Cosmetics.* Beijing: National Medicine Products Administration (2015).

[38] Jung G Tse J Guiha I Rao J. Prospective, randomized, open-label trial comparing the safety, efficacy, and tolerability of an acne treatment regimen with and without a probiotic supplement and minocycline in subjects with mild to moderate acne. *J Cutan Med Surg.* (2013) 17:114–22. 10.2310/7750.2012.12026 23582165

[39] Chen T Liu Y Huang L. ImageGP: an easy-to-use data visualization web server for scientific researchers. *iMeta.* (2022) 1:e5. 10.1002/imt2.5 PMC10989750 38867732

[40] Clark F. Agar-plate method for total microbial count. In: Black CA Evans DD White JL Ensminger LE Clark FE Dinaver RC editors. *Methods of Soil Analysis, Part 2. Chemical and Microbiological Properties.* (Madison, WI: American Society of Agronomy) (2016). p. 1460–6. 10.2134/agronmonogr9.2.c48

[41] Awan S Lu J. Management of severe acne during pregnancy: a case report and review of the literature. *Int J Womens Dermatol.* (2017) 3:145–50. 10.1016/j.ijwd.2017.06.001 28831424 PMC5555287

[42] Anderson K Dothard E Huang K Feldman S. Frequency of primary nonadherence to acne treatment. *JAMA Dermatol.* (2015) 151:623–6. 10.1001/jamadermatol.2014.5254 25793290

[43] Yentzer B Ade R Fountain J Clark A Taylor S Fleischer A Jr Simplifying regimens promotes greater adherence and outcomes with topical acne medications: a randomized controlled trial. *Cutis.* (2010) 86:103–8. 20919606

[44] Layton A Thiboutot D Tan J. Reviewing the global burden of acne: how could we improve care to reduce the burden. *Br J Dermatol.* (2021) 184:219–25. 10.1111/bjd.19477 32770673

[45] Kemény L Szabó K. Innate and adaptive immunity in acne vulgaris. In: Suh DH editor. *Acne. Updates in Clinical Dermatology.* (Cham: Springer) (2021). p. 149–57. 10.1007/978-3-030-68996-4_14

[46] Brown S Shalita A. Acne vulgaris. *Lancet.* (1998) 351:1871–6. 10.1016/S0140-6736(98)01046-0 9652685

[47] Barnard E Shi B Kang D Craft N Li H. The balance of metagenomic elements shapes the skin microbiome in acne and health. *Sci Rep.* (2016) 6:1–12. 10.1038/srep39491 28000755 PMC5175143

[48] Nagy I Pivarcsi A Koreck A Széll M Urbán E Kemény L. Distinct strains of *Propionibacterium acnes* induce selective human β-defensin-2 and interleukin-8 expression in human keratinocytes through toll-like receptors. *J Invest Dermatol.* (2005) 124:931–8. 10.1111/j.0022-202X.2005.23705.x 15854033

[49] Agak G Kao S Ouyang K Qin M Moon D Butt A Phenotype and antimicrobial activity of Th17 cells induced by *Propionibacterium acnes* strains associated with healthy and acne skin. *J Invest Dermatol.* (2018) 138:316–24. 10.1016/j.jid.2017.07.842 28864077 PMC5794628

[50] Nagy I Pivarcsi A Kis K Koreck A Bodai L McDowell A *Propionibacterium acnes* and lipopolysaccharide induce the expression of antimicrobial peptides and proinflammatory cytokines/chemokines in human sebocytes. *Microbes Infect.* (2006) 8:2195–205. 10.1016/j.micinf.2006.04.001 16797202

[51] Yu Y Champer J Agak G Kao S Modlin R Kim J. Different *Propionibacterium acnes* phylotypes induce distinct immune responses and express unique surface and secreted proteomes. *J Invest Dermatol.* (2016) 136:2221–8. 10.1016/j.jid.2016.06.615 27377696 PMC8295477

[52] Lebeer S Oerlemans E Claes I Henkens T Delanghe L Wuyts S Selective targeting of skin pathobionts and inflammation with topically applied lactobacilli. *Cell Rep Med.* (2022) 3:100521. 10.1016/j.xcrm.2022.100521 35243421 PMC8861818

[53] Rinaldi F Marotta L Mascolo A Amoruso A Pane M Giuliani G Facial acne: a randomized, double-blind, placebo-controlled study on the clinical efficacy of a symbiotic dietary supplement. *Dermatol Ther (Heidelb).* (2022) 12:577–89. 10.1007/s13555-021-00664-z 35061237 PMC8850513

[54] Oh S Kim S Ko Y Sim J Kim K Lee S Effect of bacteriocin produced by *Lactococcus* sp. HY 449 on skin-inflammatory bacteria. *Food Chem Toxicol.* (2006) 44:552–9. 10.1016/j.fct.2005.08.030 16226831

[55] Lew L Liong M. Bioactives from probiotics for dermal health: functions and benefits. *J Appl Microbiol.* (2013) 114:1241–53. 10.1111/jam.12137 23311666

[56] Kang B Seo J Lee G Kim J Kim S Han Y Antimicrobial activity of enterocins from *Enterococcus faecalis* SL-5 against *Propionibacterium acnes*, the causative agent in acne vulgaris, and its therapeutic effect. *J Microbiol.* (2009) 47:101–9. 10.1007/s12275-008-0179-y 19229497

[57] Cinque B Torre CL Melchiorre E Marchesani G Zoccali G Palumbo P Use of probiotics for dermal applications. In: Liong M editor. *Probiotics. Microbiology Monographs.* (Berlin: Springer) (2011). p. 221–41.

[58] Chen Y Fischbach M Belkaid Y. Skin microbiota–host interactions. *Nature.* (2018) 553:427–36. 10.1038/nature25177 29364286 PMC6075667

