# Supplementary material for: A pilot study on the efficacy of topical lotion containing anti-acne postbiotic in subjects with mild -to -moderate acne
Source: Front Med (Lausanne). 2022 Dec 9;9:1064460. doi: 10.3389/fmed.2022.1064460 (PMC9780477; doi:10.3389/fmed.2022.1064460)
Supplement: Supplementary file 1 [file Table_1.DOCX]

**Table S1.** Subject information

| No. | Sex | Years | Completed | Analysis |
| --- | --- | --- | --- | --- |
| S001 | Female | 21 | Yes | Yes |
| S002 | Male | 24 | Yes | Yes |
| S003 | Female | 25 | Yes | Yes |
| S004 | Male | 23 | Yes | Yes |
| S005 | Female | 25 | Yes | Yes |
| S006 | Male | 21 | Yes | Yes |
| S007 | Male | 20 | Yes | Yes |
| S008 | Female | 23 | Yes | Yes |
| S009 | Male | 21 | Yes | Yes |
| S010 | Female | 24 | Yes | Yes |
| S011 | Female | 24 | Yes | Yes |
| S012 | Male | 19 | Yes | Yes |
| S013 | Male | 24 | Yes | Yes |
| S014 | Male | 21 | Yes | Yes |
| S015 | Male | 24 | Yes | Yes |
| S016 | Male | 24 | Yes | Yes |
| S017 | Female | 21 | Yes | Yes |
| S018 | Female | 24 | Yes | Yes |
| S019 | Female | 22 | Yes | Yes |
| S020 | Female | 20 | Yes | Yes |
| S021 | Male | 21 | Yes | Yes |
| S022 | Male | 23 | Yes | Yes |

**Table S2.** Proportions of acne lesion

| No. | W0 | W1 | W2 | W3 | W4 | IRs 1 | IRs 2 | IRs 3 | IRs 4 |
| --- | --- | --- | --- | --- | --- | --- | --- | --- | --- |
| S001 | 9.0% | 11.0% | 7.0% | 6.0% | 2.0% | 22.2% | -22.2% | -33.3% | -77.8% |
| S002 | 3.0% | 0.0% | 0.0% | 3.0% | 0.0% | -100.0% | -100.0% | 0.0% | -100.0% |
| S003 | 3.0% | 0.0% | 1.0% | 1.0% | 0.0% | -100.0% | -66.7% | -66.7% | -100.0% |
| S004 | 31.0% | 11.0% | 14.0% | 7.0% | 20.0% | -64.5% | -54.8% | -77.4% | -35.5% |
| S005 | 8.0% | 0.0% | 1.0% | 0.0% | 1.0% | -100.0% | -87.5% | -100.0% | -87.5% |
| S006 | 4.0% | 3.0% | 2.0% | 1.0% | 1.0% | -25.0% | -50.0% | -75.0% | -75.0% |
| S007 | 7.0% | 1.0% | 1.0% | 5.0% | 1.0% | -85.7% | -85.7% | -28.6% | -85.7% |
| S008 | 5.0% | 1.0% | 1.0% | 2.0% | 1.0% | -80.0% | -80.0% | -60.0% | -80.0% |
| S009 | 8.0% | 8.0% | 4.0% | 4.0% | 8.0% | 0.0% | -50.0% | -50.0% | 0.0% |
| S010 | 6.0% | 0.0% | 1.0% | 4.0% | 0.0% | -100.0% | -83.3% | -33.3% | -100.0% |
| S011 | 1.0% | 3.0% | 1.0% | 2.0% | 6.0% | 200.0% | 0.0% | 100.0% | 500.0% |
| S012 | 5.0% | 5.0% | 2.0% | 3.0% | 6.0% | 0.0% | -60.0% | -40.0% | 20.0% |
| S013 | 5.0% | 11.0% | 8.0% | 8.0% | 5.0% | 120.0% | 60.0% | 60.0% | 0.0% |
| S014 | 5.0% | 3.0% | 2.0% | 2.0% | 4.0% | -40.0% | -60.0% | -60.0% | -20.0% |
| S015 | 13.0% | 9.0% | 16.0% | 3.0% | 9.0% | -30.8% | 23.1% | -76.9% | -30.8% |
| S016 | 7.0% | 2.0% | 4.0% | 1.0% | 4.0% | -71.4% | -42.9% | -85.7% | -42.9% |
| S017 | 3.0% | 1.0% | 1.0% | 1.0% | 3.0% | -66.7% | -66.7% | -66.7% | 0.0% |
| S018 | 12.0% | 39.0% | 2.0% | 4.0% | 14.0% | 225.0% | -83.3% | -66.7% | 16.7% |
| S019 | 7.0% | 8.0% | 5.0% | 4.0% | 5.0% | 14.3% | -28.6% | -42.9% | -28.6% |
| S020 | 3.0% | 1.0% | 2.0% | 3.0% | 3.0% | -66.7% | -33.3% | 0.0% | 0.0% |
| S021 | 12.0% | 12.0% | 12.0% | 13.0% | 14.0% | 0.0% | 0.0% | 8.3% | 16.7% |
| S022 | 7.0% | 7.0% | 6.0% | 6.0% | 8.0% | 0.0% | -14.3% | -14.3% | 14.3% |

W0: baseline; W1: Week 1; W2: Week 2; W3：Week 3; W4: Week 4. IRs 1: Improvement Rates at Week 1; IRs 2: Improvement Rates at Week 2; IRs 3: Improvement Rates at Week 3; IRs 4: Improvement Rates at Week 4.

**Table S3.** Pore/AOI (mm^2^)

| No. | W0 | W1 | W2 | W3 | W4 | IRs 1 | IRs 2 | IRs 3 | IRs 4 |
| --- | --- | --- | --- | --- | --- | --- | --- | --- | --- |
| S001 | 6.2% | 6.0% | 5.9% | 6.2% | 5.4% | -3.2% | -4.8% | 0.0% | -12.9% |
| S002 | 6.5% | 6.4% | 6.5% | 6.6% | 6.7% | -1.5% | 0.0% | 1.5% | 3.1% |
| S003 | 6.0% | 6.0% | 6.4% | 6.3% | 6.2% | 0.0% | 6.7% | 5.0% | 3.3% |
| S004 | 6.1% | 6.4% | 6.2% | 6.0% | 6.3% | 4.9% | 1.6% | -1.6% | 3.3% |
| S005 | 4.7% | 4.7% | 4.9% | 4.9% | 4.9% | 0.0% | 4.3% | 4.3% | 4.3% |
| S006 | 5.6% | 5.5% | 5.6% | 5.9% | 5.9% | -1.8% | 0.0% | 5.4% | 5.4% |
| S007 | 6.9% | 6.8% | 7.2% | 7.1% | 7.3% | -1.4% | 4.3% | 2.9% | 5.8% |
| S008 | 7.3% | 7.0% | 7.2% | 7.2% | 7.1% | -4.1% | -1.4% | -1.4% | -2.7% |
| S009 | 6.0% | 6.3% | 6.2% | 6.2% | 6.6% | 5.0% | 3.3% | 3.3% | 10.0% |
| S010 | 8.7% | 8.7% | 8.6% | 8.8% | 8.9% | 0.0% | -1.1% | 1.1% | 2.3% |
| S011 | 4.3% | 3.5% | 3.6% | 3.3% | 3.2% | -18.6% | -16.3% | -23.3% | -25.6% |
| S012 | 7.0% | 7.0% | 6.6% | 6.8% | 6.7% | 0.0% | -5.7% | -2.9% | -4.3% |
| S013 | 4.4% | 4.9% | 4.5% | 4.6% | 4.5% | 11.4% | 2.3% | 4.5% | 2.3% |
| S014 | 7.1% | 7.3% | 7.2% | 7.2% | 7.2% | 2.8% | 1.4% | 1.4% | 1.4% |
| S015 | 8.2% | 8.3% | 8.3% | 8.3% | 8.4% | 1.2% | 1.2% | 1.2% | 2.4% |
| S016 | 6.4% | 6.4% | 6.4% | 6.8% | 6.7% | 0.0% | 0.0% | 6.3% | 4.7% |
| S017 | 6.6% | 7.2% | 7.1% | 7.2% | 7.1% | 9.1% | 7.6% | 9.1% | 7.6% |
| S018 | 6.9% | 6.8% | 6.8% | 6.9% | 6.8% | -1.4% | -1.4% | 0.0% | -1.4% |
| S019 | 5.4% | 5.7% | 5.5% | 5.9% | 6.0% | 5.6% | 1.9% | 9.3% | 11.1% |
| S020 | 5.6% | 5.2% | 5.6% | 0.0% | 0.0% | -7.1% | 0.0% | -100.0% | -100.0% |
| S021 | 5.5% | 5.8% | 5.7% | 0.0% | 0.0% | 5.5% | 3.6% | -100.0% | -100.0% |
| S022 | 5.6% | 5.7% | 5.6% | 5.3% | 5.5% | 1.8% | 0.0% | -5.4% | -1.8% |

W0: baseline; W1: Week 1; W2: Week 2; W3：Week 3; W4: Week 4. IRs 1: Improvement Rates at Week 1; IRs 2: Improvement Rates at Week 2; IRs 3: Improvement Rates at Week 3; IRs 4: Improvement Rates at Week 4.

**Table S4.** SCH

| No. | W0 | W2 | W4 | IRs 2 | IRs 4 |
| --- | --- | --- | --- | --- | --- |
| S001 | 68.0 | 75.3 | 73.0 | 10.7% | 7.4% |
| S002 | 36.4 | 72.1 | 64.3 | 98.1% | 76.6% |
| S003 | 79.1 | 68.0 | 74.0 | -14.0% | -6.4% |
| S004 | 48.4 | 41.8 | 59.8 | -13.6% | 23.6% |
| S005 | 55.8 | 52.0 | 56.9 | -6.8% | 2.0% |
| S006 | 41.2 | 33.0 | 30.9 | -19.9% | -25.0% |
| S007 | 36.9 | 54.4 | 49.7 | 47.4% | 34.7% |
| S008 | 66.2 | 59.1 | 59.8 | -10.7% | -9.7% |
| S009 | 69.9 | 64.7 | 66.9 | -7.4% | -4.3% |
| S010 | 51.4 | 49.3 | 48.8 | -4.1% | -5.1% |
| S011 | 52.3 | 54.0 | 60.8 | 3.3% | 16.3% |
| S012 | 54.7 | 46.2 | 61.3 | -15.5% | 12.1% |
| S013 | 59.2 | 62.4 | 52.5 | 5.4% | -11.3% |
| S014 | 50.2 | 40.3 | 58.6 | -19.7% | 16.7% |
| S015 | 43.8 | 20.2 | 37.0 | -53.9% | -15.5% |
| S016 | 39.4 | 49.7 | 47.7 | 26.1% | 21.1% |
| S017 | 52.5 | 44.3 | 46.1 | -15.6% | -12.2% |
| S018 | 62.0 | 77.5 | 63.2 | 25.0% | 1.9% |
| S019 | 59.7 | 65.4 | 66.9 | 9.5% | 12.1% |
| S020 | 58.7 | 48.3 | 63.7 | -17.7% | 8.5% |
| S021 | 69.7 | 65.7 | 67.8 | -5.7% | -2.7% |
| S022 | 53.2 | 50.9 | 53.7 | -4.3% | 0.9% |

W0: baseline; W2: Week 2; W4: Week 4. IRs 2: Improvement Rates at Week 2; IRs 4: Improvement Rates at Week 4.

**Table S5.** TEWL(g/m^2^/h)

| No. | W0 | W2 | W4 | IRs 2 | IRs 4 |
| --- | --- | --- | --- | --- | --- |
| S001 | 11.9 | 11.5 | 11.4 | -3.4% | -0.9% |
| S002 | 14.7 | 12.2 | 10.1 | -17.0% | -17.2% |
| S003 | 11.3 | 13.2 | 15.3 | 16.8% | 15.9% |
| S004 | 15.9 | 13.3 | 12.3 | -16.4% | -7.5% |
| S005 | 19.6 | 17.5 | 10.7 | -10.7% | -38.9% |
| S006 | 24.7 | 20 | 19 | -19.0% | -5.0% |
| S007 | 20.5 | 13.8 | 13.9 | -32.7% | 0.7% |
| S008 | 12.1 | 11.6 | 11.4 | -4.1% | -1.7% |
| S009 | 19.6 | 15.9 | 14.2 | -18.9% | -10.7% |
| S010 | 19.1 | 15.8 | 14.4 | -17.3% | -8.9% |
| S011 | 13.8 | 12.6 | 12.7 | -8.7% | 0.8% |
| S012 | 12.3 | 14.4 | 17.8 | 17.1% | 23.6% |
| S013 | 13.7 | 13.9 | 14.9 | 1.5% | 7.2% |
| S014 | 15.5 | 15.1 | 10.1 | -2.6% | -33.1% |
| S015 | 14.8 | 19.8 | 17.6 | 33.8% | -11.1% |
| S016 | 14.1 | 12.5 | 13.5 | -11.3% | 8.0% |
| S017 | 17.7 | 13.5 | 12.4 | -23.7% | -8.1% |
| S018 | 12.3 | 12.6 | 14.7 | 2.4% | 16.7% |
| S019 | 12.8 | 15 | 10.5 | 17.2% | -30.0% |
| S020 | 21.7 | 16.3 | 11.4 | -24.9% | -30.1% |
| S021 | 12.8 | 13.1 | 13.9 | 2.3% | 8.6% |
| S022 | 15.5 | 16.5 | 16.7 | 6.5% | 7.7% |

W0: baseline; W2: Week 2; W4: Week 4. IRs 2: Improvement Rates at Week 2; IRs 4: Improvement Rates at Week 4.

**Table S6.** Sebum Contents(μg/cm^2^)

| No. | W0 | W2 | W4 | IRs 2 | IRs 4 |
| --- | --- | --- | --- | --- | --- |
| S001 | 29 | 28 | 23 | -3.4% | -17.9% |
| S002 | 24 | 22 | 16 | -8.3% | -27.3% |
| S003 | 43 | 29 | 36 | -32.6% | 24.1% |
| S004 | 35 | 9 | 22 | -74.3% | 144.4% |
| S005 | 36 | 36 | 27 | 0.0% | -25.0% |
| S006 | 82 | 85 | 86 | 3.7% | 1.2% |
| S007 | 69 | 69 | 66 | 0.0% | -4.3% |
| S008 | 48 | 42 | 46 | -12.5% | 9.5% |
| S009 | 67 | 52 | 66 | -22.4% | 26.9% |
| S010 | 20 | 24 | 20 | 20.0% | -16.7% |
| S011 | 81 | 61 | 48 | -24.7% | -21.3% |
| S012 | 25 | 51 | 37 | 104.0% | -27.5% |
| S013 | 67 | 32 | 24 | -52.2% | -25.0% |
| S014 | 98 | 93 | 93 | -5.1% | 0.0% |
| S015 | 47 | 39 | 49 | -17.0% | 25.6% |
| S016 | 51 | 43 | 75 | -15.7% | 74.4% |
| S017 | 58 | 44 | 30 | -24.1% | -31.8% |
| S018 | 46 | 14 | 14 | -69.6% | 0.0% |
| S019 | 35 | 15 | 17 | -57.1% | 13.3% |
| S020 | 57 | 44 | 39 | -22.8% | -11.4% |
| S021 | 49 | 59 | 61 | 20.4% | 24.5% |
| S022 | 80 | 73 | 73 | -8.8% | -8.8% |

W0: baseline; W2: Week 2; W4: Week 4. IRs 2: Improvement Rates at Week 2; IRs 4: Improvement Rates at Week 4.

**Table S7.** Facial skin pH

| No. | W0 | W2 | W4 | IRs 2 | IRs 4 |
| --- | --- | --- | --- | --- | --- |
| S001 | 5.6 | 5.2 | 5.3 | -7.1% | -5.4% |
| S002 | 4.9 | 4.7 | 4.5 | -4.1% | -8.2% |
| S003 | 5.6 | 5.4 | 5.6 | -3.6% | 0.0% |
| S004 | 4.2 | 5.2 | 5.1 | 23.8% | 21.4% |
| S005 | 5.4 | 5.7 | 5.5 | 5.6% | 1.9% |
| S006 | 5.5 | 5.6 | 5.8 | 1.8% | 5.5% |
| S007 | 4.9 | 5.1 | 5.2 | 4.1% | 6.1% |
| S008 | 5.8 | 5.5 | 5.5 | -5.2% | -5.2% |
| S009 | 5.3 | 5 | 5.3 | -5.7% | 0.0% |
| S010 | 5.6 | 5.9 | 5.9 | 5.4% | 5.4% |
| S011 | 5.5 | 6.3 | 5.8 | 14.5% | 5.5% |
| S012 | 6 | 5.6 | 5.8 | -6.7% | -3.3% |
| S013 | 5.9 | 5.4 | 5.7 | -8.5% | -3.4% |
| S014 | 5.3 | 5.4 | 5.9 | 1.9% | 11.3% |
| S015 | 5.3 | 5.9 | 5.6 | 11.3% | 5.7% |
| S016 | 5.3 | 6.1 | 5.4 | 15.1% | 1.9% |
| S017 | 5.7 | 5.7 | 5.9 | 0.0% | 3.5% |
| S018 | 5.2 | 5.2 | 5.2 | 0.0% | 0.0% |
| S019 | 5.3 | 5.5 | 5.5 | 3.8% | 3.8% |
| S020 | 5.5 | 5.8 | 5.9 | 5.5% | 7.3% |
| S021 | 5.7 | 5.8 | 5.7 | 1.8% | 0.0% |
| S022 | 5.4 | 5.3 | 5.5 | -1.9% | 1.9% |

W0: baseline; W2: Week 2; W4: Week 4. IRs 2: Improvement Rates at Week 2; IRs 4: Improvement Rates at Week 4.
